# Hair Mercury Levels in Pregnant Women: Fish Consumption as a Determinant of Exposure

**DOI:** 10.3390/toxics12050366

**Published:** 2024-05-16

**Authors:** Olga Rumiantseva, Viktor Komov, Mikhail Kutuzov, Hicham Zaroual, Ksenia Mizina, Maria Belova, Igor Nikitin, Alla Stolyarova, Dmitry Mashin, Daria Vilkova

**Affiliations:** 1 Research Laboratory of Applied Biotechnology, Cherepovets State University, 5 Lunacharsky pr., 162602 Cherepovets, Russia; vkomov@ibiw.ru (V.K.); kutuzov35@gmail.com (M.K.); iaa.hzaroual@gmail.com (H.Z.); ksenya8619@yandex.ru (K.M.); mabelova@chsu.ru (M.B.); nikito.igor@gmail.com (I.N.); 2 Physiology and Toxicology Laboratory, Papanin Institute for Biology of Inland Waters, Russian Academy of Sciences, 152742 Borok, Russia; 3 Sustainable AgriFoodtech Innovation & Research (SAFIR), 62000 Arras, France; 4 Applied Organic Chemistry Laboratory, Sidi Mohamed Ben Abdellah University, Fez M-30000, Morocco; 5 Research Laboratory of Nutritional Systems Biotechnology, Plekhanov Russian University of Economics, 36 Stremyanny per., 117997 Moscow, Russia; stolyarova2011@mail.ru; 6 Department of Biotechnology of Food Products from Plant and Animal Raw Materials, K.G. Razumovsky Moscow State University of Technologies and Management (the First Cossack University), 73 Zemlyanoy Val, 109004 Moscow, Russia; 7 Department of Management and Economics, State University of Humanities and Social Studies, 30 Zelenaya st., 140400 Moscow, Russia; dmashin@ya.ru

**Keywords:** mercury, pregnant women, fish consumption, hair

## Abstract

The consumption of fish in food may contain mercury, a harmful element and dangerous chemical detrimental to human health. The purpose of this study was to determine the mercury level in the hair of pregnant women with different fish intakes in their diets. The concentration of total mercury in hair was determined using an atomic absorption spectrometer. In this study, 98 pregnant women were invited to participate (aged from 18 to 48 years). The mean content of mercury in the hair of pregnant women in Northwestern Russia was 0.428 mg/kg (ranging from 0.018 to 3.1 mg/kg). As a result, 22% of women had mercury values above 0.58 mg/kg, which is considered dangerous for the fetus. The hair mercury concentration in a village area was higher than that in a city area (i.e., 0.548 mg/kg and 0.326 mg/kg). Moreover, the maximum level of mercury was noted for a group of pregnant women who consumed more than 5 kg/month of fish and fish products. Furthermore, the consumption of freshwater fish in the diet leads to a higher mercury content in the hair of pregnant women than the consumption of marine fish.

## 1. Introduction

The government has increasingly focused on public health, particularly in the past decade. Fish and fish products are gaining popularity worldwide due to being natural sources of easily digestible protein, as well as containing essential micronutrients, vitamins, and polyunsaturated fatty acids (omega-3), making them a recommended component of a balanced diet [1]. Indeed, the fishery and aquaculture industry has experienced remarkable growth over the past few decades, leading to record levels of total production, market expansion, and fish consumption [2]. By 2018, the total world capture fisheries reached a record level of 179 million tons, for which 156 million tons were destined for human consumption. Consequently, fish consumption significantly increased from 9.0 kg per capita in 1961 to 20.5 kg in 2018 [3]. However, food fish may contain mercury (Hg), a potentially harmful element that is currently one of the top three most dangerous chemicals for human health [4,5,6]. Indeed, according to the United States Environmental Protection Agency, fish is the most significant source of mercury [7].

It is widely known that mercury is present in the environment primarily as forms of metallic mercury, inorganic mercury, and methylmercury. Methylmercury (MeHg) is the most dangerous form of mercury for humans since it can be efficiently transferred along the trophic web [8,9]. Approximately 90% of MeHg in total mercury (THg) is found in fish muscles [7], so MeHg is mainly responsible for exposure to mercury through fish consumption. The hair of fish consumers contains not only a higher concentration of total Hg but also a much higher fraction of methylmercury (with MeHg accounting for 70–80% of hair THg) compared with non-fish consumers, indicating that this species is responsible for exposure to Hg through food consumption [10]. Increased exposure to mercury significantly heightens the risk of cardiovascular diseases, including hypertension, diabetes, and metabolic syndrome, in humans [11,12,13]. People living near water are at high risk of exposure to mercury, as their diets contain fish and fish products [14,15]. For example, fish with elevated levels of mercury in muscles exceeding the maximum allowable concentrations have often been recorded in recent decades in the northwest of European Russia [16,17]. Rumiantseva et al. [18] observed elevated mercury levels in the hair of the indigenous rural population residing in the coastal area of the Vologda Region in Northwest Russia compared with the urban population of an industrial city. The authors presented their findings concerning women of childbearing age, adults, and children during the period from 2016 to 2022 [19]. Nevertheless, there is no information on mercury content in pregnant women’s hair in the Vologda Region. Exposure to mercury is especially dangerous for pregnant women since there is a risk of the transmission of Hg from mother to fetus due to there being no preventive barrier [20]. Kobayashi et al. [21] pointed out, in their investigations, that prenatal exposures to mercury have been correlated with low birth weight and premature birth and can cause developmental problems in children. Currently, the acceptable levels of mercury in hair proposed by various organizations are different. Indeed, the US EPA has established recommended Hg levels of 1 mg/kg or less in hair for humans [7]. The highest level was proposed by the Food and Agriculture Organization and the World Health Organization as 2.3 mg/kg of Hg in hair [22]. Moreover, some authors consider a 0.58 mg/kg mercury level for women of reproductive age to increase the risk to the fetus [23]. Several investigations have shown different levels of mercury in the hair of pregnant women: 0.48 mg/kg to 3.52 mg/kg for Indonesia and Iran, respectively [24,25]. Thus, monitoring mercury content is especially important for pregnant women to prevent the risks of prenatal Hg exposure in the fetus and is recommended for controlling concentrations of mercury.

However, to the best knowledge of the authors, research on hair mercury levels in pregnant women depending on fish consumption as a determinant of exposure in Russia has not been investigated to date. The purpose of this study was to determine the mercury levels in the hair of pregnant women from two areas of Northwest Russia (Vologda Region), Cherepovets City and Vokhtoga Village. In this context, the results of this study will contribute to the development of an assessment system for mercury exposure among pregnant women and will allow us to provide a model for monitoring different groups of people based on the example of the Vologda Region in the future.

## 2. Materials and Methods

### 2.1. Sampling Location and Research Subjects

**Figure 1:**
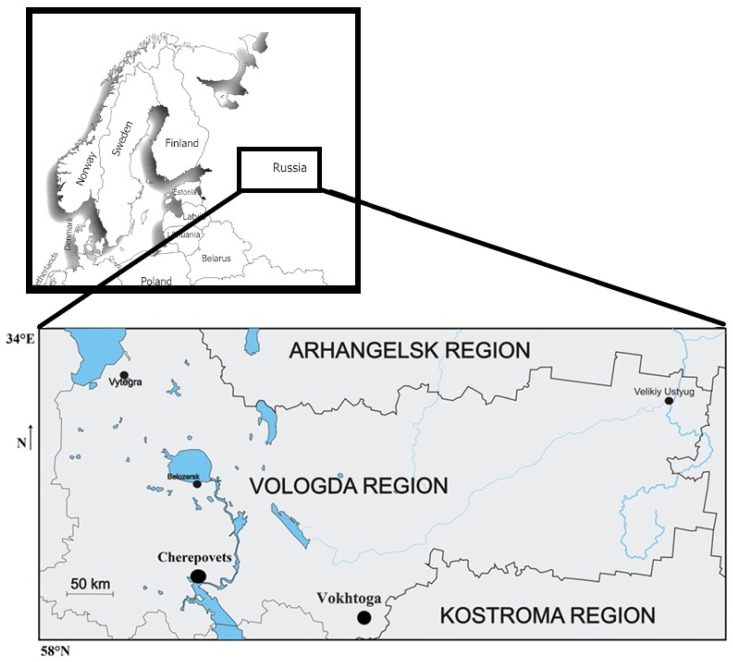
The region of study, showing the Cherepovets City area and the Vokhtoga Village area.

The sampling location was in the northwest of the European part of Russia. This study was conducted on 98 pregnant women aged from 18 to 48 years, of which 53 people were from Cherepovets City and 45 people were from Vokhtoga Village. Cherepovets City and Vokhtoga Village are located in the Vologda Region (Figure 1).

Cherepovets is located in the west of the region on the banks of the Sheksna River (a tributary of the Volga River) and on the shores of the Rybinsk Reservoir. Vokhtoga is located on the low slope of the watershed of the Monza and Lezha Rivers, on the right bank of the Vokhtozhka River.

All subjects gave their informed consent for inclusion before they participated in the study. This study was conducted in accordance with the Declaration of Helsinki, and the protocol was approved by Ethics Committee No. 2-1/55. The main criteria for participants were as follows: aged more than 18 years and the presence of pregnancy. Measurements were performed in accordance with the World Medical Association (WMA) Declaration of Helsinki: Ethical Principles for Medical Research Involving Human Subjects [26]. The Bioethics Commission of Cherepovets State University and the Territorial Department of Health of the Vologda Region (No. 2-1/55) discussed and approved this investigation.

The sampling process was conducted in 2022. Each pregnant woman completed a questionnaire to indicate their age, place of residence (city area or village area), the predominant type of fish in the diet (does not eat fish, sea fish, or freshwater fish), and the amount of fish consumed (none, up to 1 kg/year, up to 1 kg/month, or up to 5 kg/month)

### 2.2. Measurement of Hair Mercury

Hair strands were collected by cutting hair from the occipital region of the scalp according to the WHO recommendations [27]. The mercury content was determined in hair from the root of about 2 cm long. The samples were stored in a polyethylene bag and stapled at the proximal end to keep the hair sample together until the Hg measurements. The concentration of mercury in human hair was determined using the atomic absorption method without any preliminary sample preparation on dry hair. A portion weighing 20–40 mg of the prepared sample was placed in an RA-915M mercury analyzer equipped with a PYRO-915+ pyrolysis unit (Lumex Ltd., St. Petersburg, Russia), wherein the thermal decomposition of the sample occurs with the simultaneous atomization of mercury. The quantitative determination of total mercury was carried out by atomic absorption spectrometry with Zeeman correction of non-selective absorption. The samples were analyzed at the Regional Shared Services Center of Cherepovets State University. Before each measurement, a certified reference material (National Institute for Minamata Disease—NIMD-1 Human Hair, Japan) was recorded and used as quality control. The average mercury concentration was 0.794 ± 0.05 mg/kg. The equipment was monitored every 30 measurements (relative percent difference (RPD) < 20%).

### 2.3. Statistical Analysis

The Mann–Whitney U test was used to compare the mean Hg concentration among two independent groups of pregnant women (in both areas); for three or more independent groups, the Kruskal–Wallis test was applied (the amount of fish consumed; the predominant type of fish in the diet) for a *p*-value less 0.05. In this article, the data are presented as mean ± standard error.

## 3. Results

The mean content of mercury in the hair of pregnant women was 0.428 ± 0.04 mg/kg, ranging from 0.018 to 3.1 mg/kg in the Vologda Region (Northwest Russia) (Table 1).

A correlation was established between the amount of mercury and the age of pregnant women (Rs = 0.354; *p* < 0.001), while for women in the village area, the correlation was Rs = 0.366 and *p* = 0.013; for women in the city area, there was no correlation (Rs = 0.053, *p* = 0.708).

**Figure 2:**
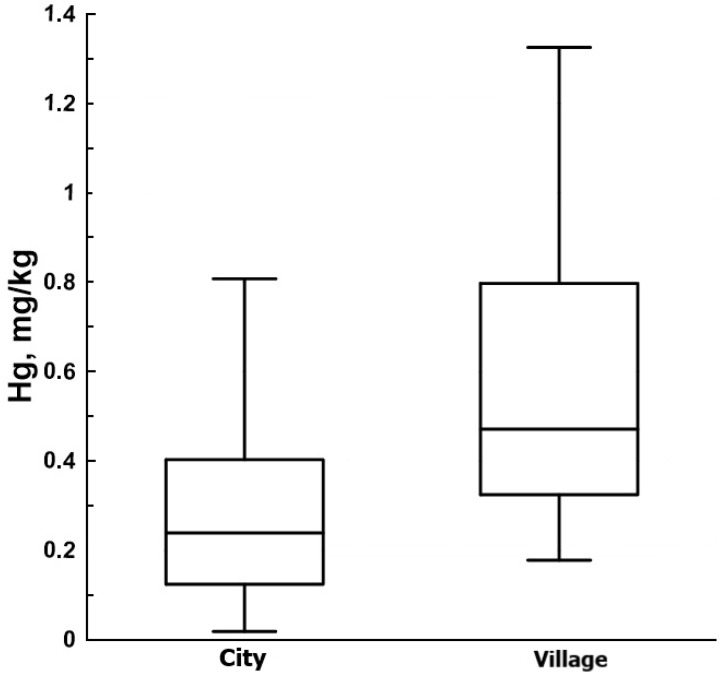
Mercury concentration in the hair of women in the city and village areas. The horizontal lines in the boxes indicate the 25th, 50th, and 75th percentiles. The top and bottom whiskers indicate the 1.5 interquartile range.

Significantly higher Hg concentrations were noted in the hair of women from the village area (0.548 ± 0.07 mg/kg) compared with women from the city area (0.326 ± 0.04 mg/kg) (Table 1, Figure 2).

**Figure 3:**
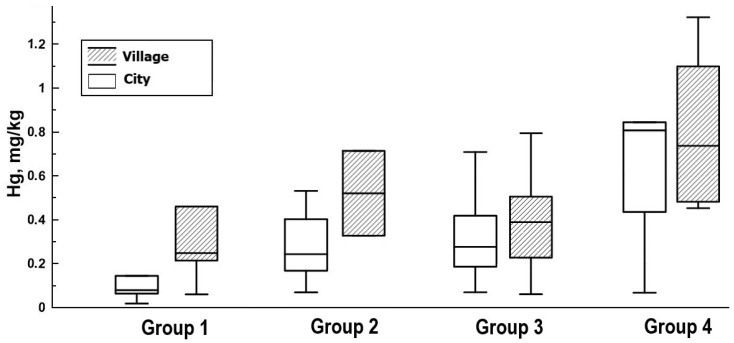
Mercury concentration in the hair of women in urban and rural areas with different amounts of fish consumed. Note: group 1—do not consume fish, group 2—up to 1 kg/year, group 3—up to 1 kg/month, and group 4—5 kg/month or more. The horizontal lines in the boxes indicate the 25th, 50th, and 75th percentiles. The top and bottom whiskers indicate the 1.5 interquartile range.

All pregnant women were divided into four groups according to the amount of fish consumed: group 1—do not consume fish, group 2—up to 1 kg/year, group 3—up to 1 kg/month, and group 4—5 kg/month or more. Differences in mercury content in women’s hair according to the frequency of fish consumption were found at a significance level of *p* < 0.05 (Table 1). The minimum value is noted in the hair of women who do not eat fish (0.236 ± 0.05 mg/kg) and eat up to 1 kg/year (0.312 ± 0.04 mg/kg); intermediate values are noted in the hair of women who eat up to 1 kg fish/month (0.37 ± 0.03 mg/kg). Maximum concentrations were observed in the hair of women with fish consumption of up to 5 kg/month (0.929 ± 0.17 mg/kg). This pattern was noted for both women from the city area and women from the village area (Figure 3).

**Figure 4:**
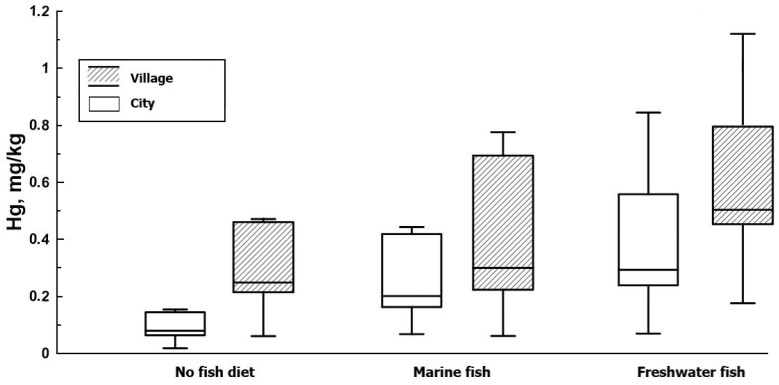
Mercury concentration in the hair of women in urban and rural areas with the predominant type of fish in the women’s diets. The horizontal lines in the boxes indicate the 25th, 50th, and 75th percentiles. The top and bottom whiskers indicate the 1.5 interquartile range.

In this study, the predominant type of fish in the women’s diets was assessed. All pregnant women were divided into three groups according to their diets: (i) 21 women who do not eat fish; (ii) 28 women with marine fish predominant in their diets; (iii) 49 women with freshwater fish predominant. Minimum concentrations of mercury (*p* < 0.05) in hair were recorded in women who did not eat fish (0.236 ± 0.05 mg/kg) and with a diet of predominantly marine fish (0.308 ± 0.04 mg/kg) (Table 1). Statistically significant high concentrations were observed in the hair of women whose diets were dominated by freshwater fish (0.579 ± 0.07 mg/kg) (Figure 4).

## 4. Discussion

**Figure 5:**
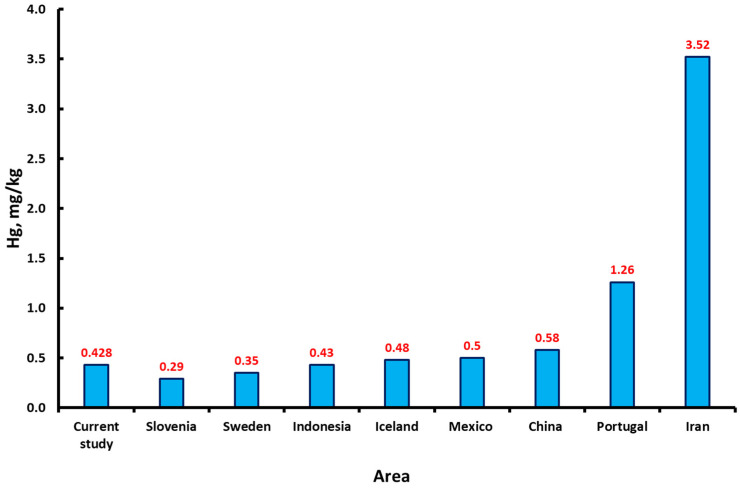
The mercury content in the hair of pregnant women in different countries (areas).

The data obtained (0.428 mg/kg) were in accordance with those of Rumiantseva et al. (2022) [28], who obtained similar results for residents (1135 women) of the Vologda Region, Russia—0.433 mg/kg—and similar to hair mercury levels in pregnant women in Mexico City (0.5 mg/kg) [29], Iceland [30] (0.48 mg/kg), Indonesia (0.43 mg/kg) [24], and China (0.58 mg/kg) [20]. Lower values have been observed in Slovenia (0.29 mg/kg) [31] and Sweden (0.35 mg/kg) [32]. A higher value was observed in research studies regarding the amount of Hg noted for pregnant women from Iran (3.52 mg/kg) [25] and Portugal (1.26 mg/kg) [33] (Figure 5).

A correlation between mercury content and age was established for the general sample and for women from the village area. The dependence of mercury accumulation from age has been noted in many studies [15,28]; the lack of dependence in the urban population is possible due to the small age range among pregnant women (18–36 years).

The mercury content in the hair of pregnant women was statistically significantly high compared with women from the city. This difference may be due to social aspects, low income in particular, which affects the diet (~78% of women from the village consume ≥1 kg/year of fish, whereas only ~47% of women from the city consume ≥1 kg/year of fish). It is widely known that the main source of protein for villages is fish protein from local reservoirs [34,35]. For city residents, with a higher level of income relative to village areas, the source of protein is not only fish but also other animal products (beef, pork, and chicken).

In this study, the share of pregnant women with mercury content above 0.58 mg/kg, which may lead to perinatal risk, was 22% from 98 participants, among them, 53 city women and 45 village women. According to research conducted in 17 European countries, 0.58 mg/kg of mercury in the hair of women could cause mild deviations in the intellectual development of children and a number of other disorders [23].

About 6% of the 98 pregnant women had values higher than the recommended standards of the US Environmental Protection Agency (1 mg/kg Hg) [7]: 5 women in the village area compared with 1 woman in the city area. One participant had a value of 3.1 mg/kg of mercury, which exceeds 2.3 mg/kg, the level recommended by the World Health Organization [22].

According to our results, the maximum concentrations of mercury were found in the hair of pregnant women who consumed up to 5 kg of fish per month. Fish used as food may be a source of mercury in human health. Statistical data on the degree of mercury load were obtained from a survey of the population, in whose diets fish traditionally form the basis [36].

For Russia, it is noted that seawater fish generally contain methylmercury (MeHg) concentrations comparable to freshwater fish [17]. However, in the study areas, high concentrations of mercury were noted in the hair of women whose diets were dominated by freshwater fish. Freshwater fish were sourced primarily from local rivers that previously had high levels of mercury. Indeed, mercury content for the main fishery species in the Vologda Region has been determined for pike (0.25 mg/kg), perch (0.21 mg/kg), and roach (0.14 mg/kg) [16]. Studies conducted in the territory of the Northwestern Federal District of the Russian Federation determined the mercury content in consumed marine fish as follows: cod (0.034 mg/kg) and trout (0.065 mg/kg). In seafood products, it is as follows: shrimp (0.013 mg/kg) and squid (0.021 mg/kg) [17]. In the group of women with a diet of predominantly freshwater fish species, mercury content exceeding 0.58 mg/kg was noted (sixteen women), of which five participants had an excess of 1 mg/kg and one woman exceeded the threshold of 2.2 mg/kg. This is mainly due to the fact that the mercury content in freshwater fish is high compared with marine fish and seafood products.

## 5. Conclusions

Mercury content was high in the hair of pregnant women from the village area compared with city area women, which was determined based on social factors and diet. The main source of mercury accumulation in the hair of pregnant women was freshwater fish. Maximum mercury values were observed in the hair of pregnant women with fish consumption of up to 5 kg/month (0.929 ± 0.17 mg/kg). Monitoring the mercury content in the hair of women is important in preventing the risks of prenatal Hg exposure on the fetus and is recommended to control concentrations of mercury, especially during pregnancy planning. For women of reproductive age, special attention must be paid to the consumption of freshwater fish in the diet, in particular from local freshwater reservoirs.

## Figures and Tables

**Table 1:** Mercury content in the hair of pregnant women in the city area and village area.

	N	Mean	Median	Min	Max	Q25	Q75	SD	SE	*p*-Value
Both areas	98	0.428	0.306	0.018	3.100	0.178	0.517	0.423	0.043	
City area	53	0.326	0.239	0.018	2.065	0.124	0.403	0.326	0.045	**a**
Village area	45	0.548	0.455	0.063	3.100	0.253	0.696	0.491	0.073	**b**
**The predominant type of fish in the diet**										
both area										
Does not eat fish	21	0.236	0.145	0.018	0.834	0.076	0.324	0.229	0.050	**a**
Sea fish	28	0.308	0.228	0.063	0.808	0.147	0.439	0.220	0.041	**a**
Freshwater fish	49	0.579	0.464	0.070	3.100	0.278	0.684	0.515	0.074	**b**
City area										
Does not eat fish	13	0.181	0.080	0.018	0.771	0.064	0.145	0.216	0.060	**a**
Sea fish	16	0.265	0.201	0.068	0.808	0.147	0.377	0.191	0.048	**a**
Freshwater fish	24	0.446	0.293	0.070	2.065	0.237	0.545	0.403	0.082	**b**
Village area										
Does not eat fish	8	0.327	0.251	0.063	0.834	0.214	0.394	0.234	0.083	**a**
Sea fish	12	0.366	0.302	0.063	0.778	0.168	0.575	0.250	0.072	**a**
Freshwater fish	25	0.706	0.506	0.178	3.100	0.455	0.797	0.582	0.116	**b**
**The amount of fish consumed**										
both area										
None	21	0.236	0.145	0.018	0.834	0.076	0.324	0.229	0.050	**a**
Up to 1 kg/year	17	0.312	0.275	0.070	0.717	0.216	0.403	0.171	0.042	**a**
Up to 1 kg/month	43	0.370	0.297	0.063	1.123	0.222	0.506	0.230	0.035	**a**
Up to 5 kg/month	17	0.929	0.778	0.068	3.100	0.478	1.074	0.716	0.174	**b**
City area										
None	13	0.181	0.080	0.018	0.771	0.064	0.145	0.216	0.060	**a**
Up to 1 kg/year	15	0.283	0.244	0.070	0.532	0.168	0.403	0.145	0.037	**ab**
Up to 1 kg/month	20	0.324	0.277	0.070	0.776	0.182	0.400	0.201	0.045	**ab**
Up to 5 kg/month	5	0.844	0.808	0.068	2.065	0.436	0.845	0.752	0.336	**b**
Village area										
None	8	0.327	0.251	0.063	0.834	0.214	0.394	0.234	0.083	**a**
Up to 1 kg/year	2	0.523	0.523	0.330	0.717	0.330	0.717	0.273	0.193	**ab**
Up to 1 kg/month	23	0.410	0.392	0.063	1.123	0.230	0.508	0.250	0.052	**a**
Up to 5 kg/month	12	0.965	0.739	0.455	3.100	0.481	1.088	0.731	0.211	**b**

Note: N—number of hair samples. mean—average value. median—median value. SD—standard deviation. SE—standard error of the mean. Min—minimum values. Max—maximum values. Q25—lower quartiles. Q75—upper quartile. a,b—letter indexes indicate statistically significant differences (*p* ≤ 0.05) according to the median test.

## Data Availability

All data are presented within the article.
